# The Potential Functional Roles of NME1 Histidine Kinase Activity in Neuroblastoma Pathogenesis

**DOI:** 10.3390/ijms21093319

**Published:** 2020-05-07

**Authors:** Kevin Adam, Jacqueline Lesperance, Tony Hunter, Peter E. Zage

**Affiliations:** 1 Molecular and Cell Biology Laboratory, Salk Institute for Biological Studies, 10010 N Torrey Pines Road, La Jolla, CA 92037, USA; kadam@salk.edu (K.A.); hunter@salk.edu (T.H.); 2 Department of Pediatrics, Division of Hematology-Oncology, University of California San Diego, La Jolla, CA 92093, USA; jlesperance@health.ucsd.edu

**Keywords:** neuroblastoma, NME/NM23/NDPK, histidine, phosphorylation, kinase

## Abstract

Neuroblastoma is the most common extracranial solid tumor in childhood. Gain of chromosome 17q material is found in >60% of neuroblastoma tumors and is associated with poor patient prognosis. The *NME1* gene is located in the 17q21.3 region, and high *NME1* expression is correlated with poor neuroblastoma patient outcomes. However, the functional roles and signaling activity of NME1 in neuroblastoma cells and tumors are unknown. NME1 and NME2 have been shown to possess histidine (His) kinase activity. Using anti-1- and 3-pHis specific monoclonal antibodies and polyclonal anti-pH118 NME1/2 antibodies, we demonstrated the presence of pH118-NME1/2 and multiple additional pHis-containing proteins in all tested neuroblastoma cell lines and in xenograft neuroblastoma tumors, supporting the presence of histidine kinase activity in neuroblastoma cells and demonstrating the potential significance of histidine kinase signaling in neuroblastoma pathogenesis. We have also demonstrated associations between NME1 expression and neuroblastoma cell migration and differentiation. Our demonstration of NME1 histidine phosphorylation in neuroblastoma and of the potential role of NME1 in neuroblastoma cell migration and differentiation suggest a functional role for NME1 in neuroblastoma pathogenesis and open the possibility of identifying new therapeutic targets and developing novel approaches to neuroblastoma therapy.

## 1. Introduction

Neuroblastoma is a pediatric tumor derived from primordial neural crest cells and is the most common extracranial solid tumor in childhood, accounting for 10% of all pediatric malignancies but more than 15% of deaths from cancer in children. Neuroblastoma is a cancer primarily of small children and is the most common form of cancer in infants, with the median age of children at the time of diagnosis being 19 months. Based on a number of clinical patient features and biological and cytogenetic tumor features, children with neuroblastoma can be divided into risk groups, and while outcomes for children with low- and intermediate-risk neuroblastoma are quite good, children with high-risk neuroblastoma, the most common and aggressive form, have extremely poor outcomes, with long-term survival rates currently less than 40% despite intensive, multimodal treatment regimens [1].

Gain of chromosome 17q material is found in greater than 60% of neuroblastoma tumors [2] and is associated with more aggressive neuroblastoma tumors and other markers of poor patient prognosis [3,4]. However, the specific gene or genes in this chromosomal region that may promote oncogenic signaling and that may represent therapeutic targets have not been conclusively identified. The gene for NME1 (also known as *NM23*, *NDPK-A*) is located in the chromosome 17q21 region commonly amplified in neuroblastoma tumors [5,6], suggesting a potential oncogenic role in neuroblastoma pathogenesis.

NME1 has been shown to be involved in multiple critical cellular behaviors, including cell proliferation, differentiation, and neural development [7,8,9]. Low *NME1* expression is correlated with poor survival and high-risk features in patients with many types of adult cancer [10,11,12,13,14], and low *NME1* expression has been found in metastatic sites of adult cancers [15,16,17,18,19]. *NME1* expression is associated with regulation of genes correlated with adult cancer metastases [20], and NME1 depletion enhances tumor metastases in xenograft models [21,22], suggesting a role for NME1 as a suppressor of metastasis. In contrast to these adult tumors, elevated *NME1* expression correlates with aggressive neuroblastoma tumor features [23,24,25] while increased *NME1* expression has been identified as a component of gene expression, signatures most significantly associated with poor neuroblastoma patient outcomes [26]. However, the functional roles of NME1 in neuroblastoma pathogenesis have not been defined.

The NME protein family consists of 10 members in human cells, and NME family members have been shown to have a variety of diverse activities, including nucleoside diphosphate kinase (NDPK) activity, geranyl/farnesyl pyrophosphate kinase activity, and exonuclease activity. The gene for human NME2 is adjacent to the *NME1* gene in the amplified chromosome 17q region, and human NME1 and NME2 share 88% sequence homology and, thus, have similar structural and functional attributes. Both NME1 and NME2 have been found to have histidine kinase activity, catalyzing transfer of the activated phosphate from the autophosphorylated histidine 118 residue (H118) onto target proteins [27]. Although histidine phosphorylation is widely used for bacterial signal transduction, NME1 and NME2 remain the only characterized histidine kinases in higher eukaryotes [28].

This work demonstrates the presence of phosphorylated histidine in neuroblastoma cells and tumors and explores the specific roles of NME1 expression in neuroblastoma pathogenesis. Ultimately, this work suggests that histidine kinases and intracellular signaling potentially regulated by histidine phosphorylation represent potential therapeutic targets in neuroblastoma.

## 2. Results

### 2.1. NME1 Expression Is Associated with Neuroblastoma Patient Outcomes and Prognostic Features

**Figure 1:**
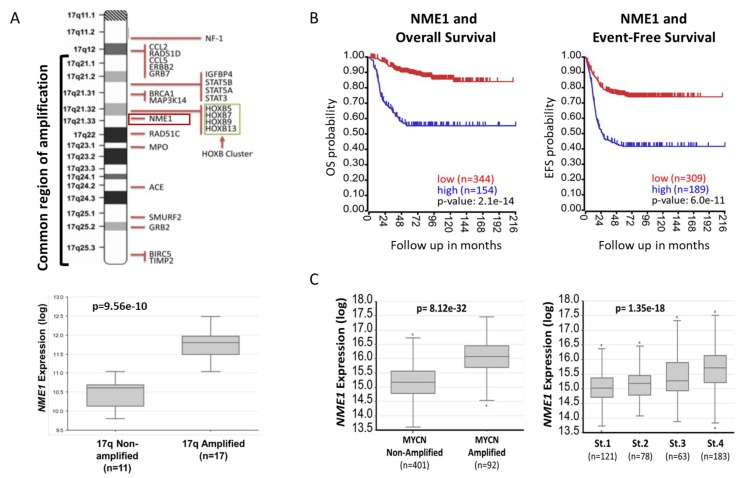
NME1 in neuroblastoma: (**A**) The chromosome 17q21 region amplified in neuroblastoma tumors is shown, with the *NME1* gene located within the amplified region (top) [29]. Relative *NME1* expression levels were plotted in patients with tumors with and without chromosome 17q amplification (bottom) using the neuroblastoma Lastowska patient dataset (*p* value = 9.56e-10) in the R2 Genomics Analysis and Visualization Platform (http://r2.amc.nl) [30]. (**B**) Using the SEQC patient dataset in the R2 Genomics Analysis and Visualization Platform, patients were divided into high (blue) and low (red) *NME1* gene expression groups and survival curves were generated. Overall survival (OS; left) and event-free survival (EFS; right) are shown with respective *p* values of 2.1e-14 and 6.0e-11 and patient numbers in parentheses. (**C**) Relative *NME1* expression levels from the SEQC patient dataset were plotted in patients with *MYCN* amplified and non-amplified tumors (*p* value = 8.12e-32) and in patients with stage 1, 2, 3, and 4 tumors (*p* value = 1.35e-18), respectively, with patient numbers shown in parentheses. The clinical characteristics of the 498 neuroblastoma patients included in Figure 1B,C are the following: Age (<18 months: 300 patients, >18 months: 198 patients); Sex (278 males, 205 females and 15 N.A). For more information, the full details of this cohort have been previously published and are available through the R2 platform [31].

The *NME1* gene is located within the chromosome 17q21 region commonly amplified in neuroblastoma tumors, and NME1 expression is highest in tumors with chromosome 17q amplification (Figure 1A), suggesting a potential oncogenic role. Expression of *NME1* is strongly associated with neuroblastoma patient outcomes, with elevated *NME1* expression associated with reduced overall and event-free survival and with the strongest associations of any of the *NME* family member genes (Figure 1B and Supplementary Data 1). *NME1* expression is also higher in tumors with *MYCN* oncogene amplification and in tumors from patients with stage 4 disease, consistent with its association with more aggressive neuroblastoma tumors (Figure 1C).

Recent work has identified lysine-histidine-pyrophosphate phosphatase (LHPP) as a histidine phosphatase and as a tumor suppressor in liver cancer [32], and elevated *LHPP* expression is associated with improved neuroblastoma patient outcomes (Supplementary Data 2), further supporting a role for the regulation of histidine phosphorylation and for histidine kinase signaling in the pathogenesis of neuroblastoma.

### 2.2. Histidine Phosphorylation in Neuroblastoma Cells and Tumors

**Figure 2:**
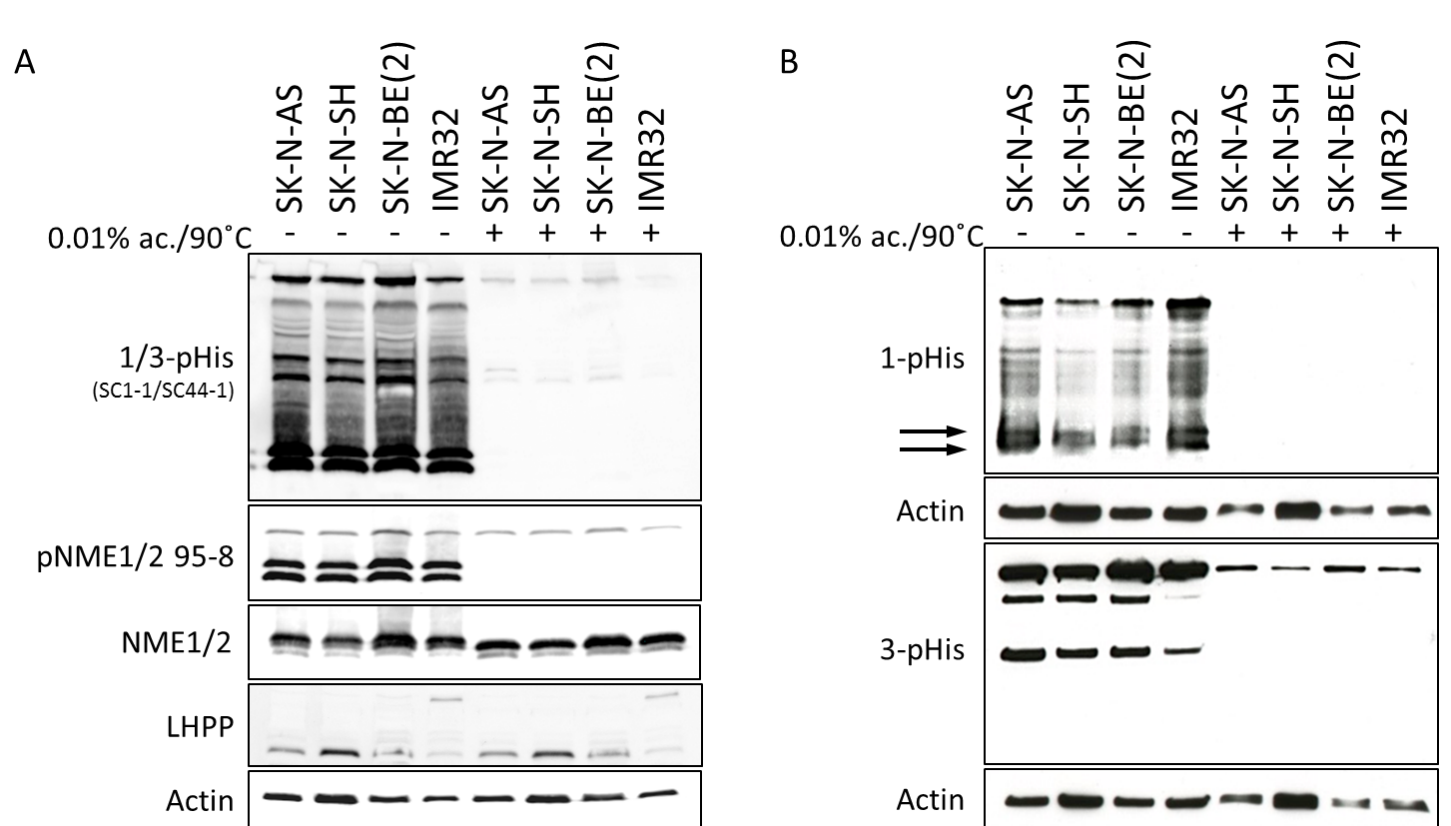
Histidine phosphorylation in neuroblastoma cell lines: (**A**) Immunoblots of neuroblastoma cell lysates were performed with a mixture of anti-1-pHis (phosphorylated histidine) and anti-3-pHis antibodies [28] and with antibodies to phosphorylated His118 (pNME1/2 95-8) (upper panels), comparing conditions where pHis is preserved (pH 10, 4 °C) and where it is lost (pH 3 (0.01% acetic acid), 90 °C). The levels of total NME1/2, lysine-histidine-pyrophosphate phosphatase (LHPP), and actin in each sample were evaluated by immunoblotting with specific antibodies (lower panels). (**B**) Immunoblots of neuroblastoma cell lysates were performed with anti-1-pHis and anti-3-pHis antibodies separately, with immunoblots for actin used as a control. Arrows indicate 1- phosphorylated histidine in NME1/NME2.

Immunoblot screening of a panel of established human neuroblastoma cell lines demonstrated both histidine phosphorylation of NME1 and NME2 (Figure 2A) and phosphorylated histidine residues on a number of other unidentified proteins, with phosphorylation at both the 1 (1-pHis) and 3 (3-pHis) isomer sites, for which the conformations have been illustrated previously [33]. As expected with histidine phosphorylation, treatment of the lysates with heat and acid resulted in loss of the histidine phosphorylation, with no effects on control proteins or total NME1/2 levels (Figure 2A,B). Furthermore, histidine phosphorylation appeared to be primarily 1-pHis, with distinct patterns of phosphorylation observed at the 1-pHis and 3-pHis sites (Figure 2B). While SK-N-BE(2) cells, which have amplification of chromosome 17q, appeared to express the highest levels of NME1/2 protein, there did not appear to be an association between relative histidine phosphorylation and expression of the histidine phosphatase LHPP.

**Figure 3:**
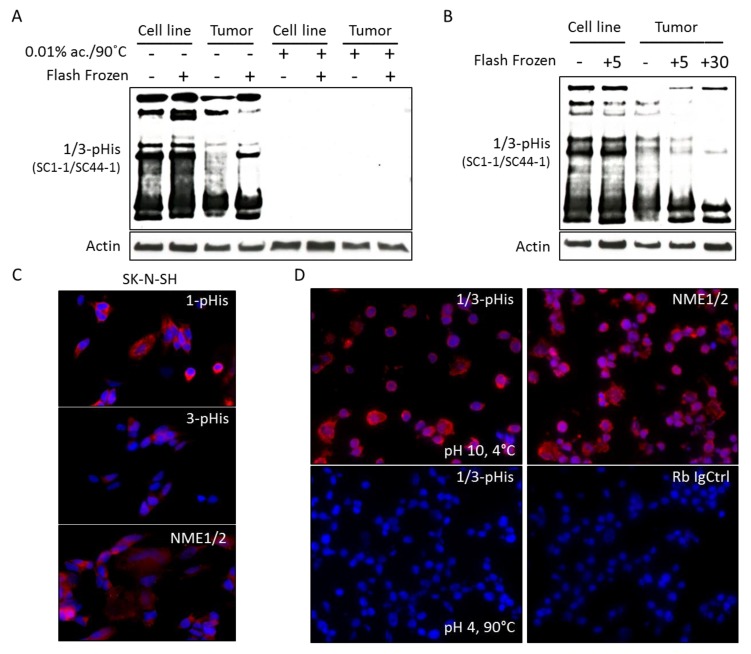
Histidine phosphorylation in neuroblastoma cell lines and tumor samples. (**A**) Immunoblots of neuroblastoma cell lysates and xenograft tumor lysates generated immediately after harvesting or after freezing in liquid N_2_ for 1/3-pHis and actin are shown, comparing conditions where pHis is preserved (pH 10, 4 °C) and where it is lost (pH 3 (0.01% acetic acid), 90 °C). (**B**) Immunoblots of neuroblastoma cell lines lysed immediately after harvesting or after a 5-min delay (+5) and of orthotopic neuroblastoma tumor samples lysed immediately after harvesting or after 5 (+5) and 30 (+30) minute delays were performed with antibodies to 1/3-pHis and actin. (**C**) Immunofluorescence images using antibodies to 1/3-pHis and total NME1/2 labeled with Alexa Fluor 568 (red) in SK-N-SH neuroblastoma cells are shown. (**D**) Immunofluorescence images using antibodies to 1/3-pHis and NME1/2 in sections of SK-N-SH neuroblastoma cells from the optimal cutting temperature (OCT) block. Combined 1- and 3-pHis, NME1/2 and rabbit IgG (Rb IgG Ctrl) were tested, comparing conditions where pHis is preserved (pH 10, 4 °C) and where it is lost (pH 4, 90 °C).

In order to confirm the presence of NME1 and of histidine phosphorylation in human neuroblastoma tumors, we analyzed mouse orthotopic xenograft tumors by immunoblot. Using 1-pHis and 3-pHis antibodies, tumor tissues were found to have persistent histidine phosphorylation of multiple proteins, including NME1/2, using fresh tumor samples as well as tumor samples that had been flash frozen in liquid nitrogen up to 30 min after removal, although the intensity of histidine phosphorylation clearly diminishes with longer time intervals prior to freezing (Figure 3A,B).

To evaluate the utility of immunofluorescence staining to detect NME1 and histidine phosphorylation in neuroblastoma cell lines, neuroblastoma cells were plated on slides or frozen in suspension at the optimal cutting temperature (OCT). Immunofluorescence using antibodies to NME1/2 and 1-pHis and 3-pHis demonstrated NME1 expression and phosphorylated histidine residues in plated cell lines and in sections of the OCT cryo-block (Figure 3C,D). The majority of the phosphorylated histidine residues appear to reside in the cytoplasm along with NME1/2 (Figure 3C,D).

### 2.3. NME1 Depletion Impacts Migration and Differentiation but not Proliferation in Neuroblastoma Cells

**Figure 4:**
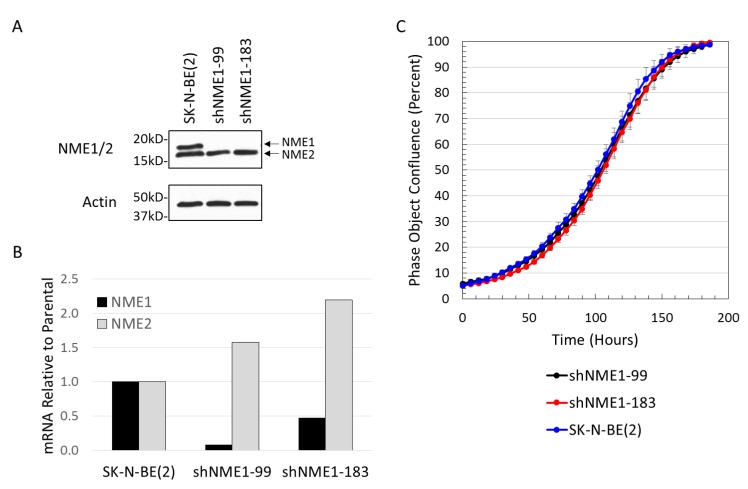
NME1 depletion in neuroblastoma cells. (**A**) SK-N-BE(2) neuroblastoma cells were transfected with shRNA’s directed against *NME1* (shNME1-99, shNME1-183) or with control shRNA (mock) and analyzed by Western blot (**A**) and Q-PCR (**B**) for NME1 and NME2 protein and gene expression, respectively. (**C**) Control SK-N-BE(2) neuroblastoma cells and cells with depleted NME1 (shNME1-99, shNME1-183) were plated and analyzed for cell confluence over time using continuous live cell imaging.

To determine the effects of altered NME1 expression on neuroblastoma cell behavior, SK-N-BE(2) neuroblastoma cell lines with shRNA-depleted NME1 were generated, resulting in cell lines with reduced NME1 gene and protein expression and an increase in *NME2* gene expression (Figure 4A,B). Selective NME1 depletion had no apparent effect on cell growth, with no observed change in the time taken to reach cell confluence (Figure 4C).

**Figure 5:**
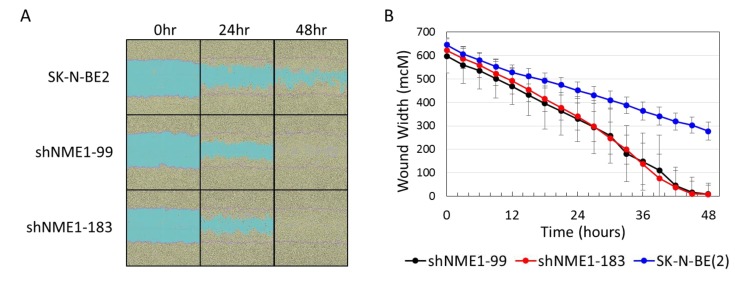
NME1 depletion affects neuroblastoma cell migration. (**A**) Control SK-N-BE(2) neuroblastoma cells and cells with depleted NME1 (shNME1-99, shNME1-183) were plated and analyzed for migration into a scratch wound using continuous live cell imaging. Images were taken every six hours and wound width was measured at each time point. Representative images (**A**) and a graph of relative wound width over time (**B**) are shown. Respective *p* values of depleted NME1 with shNME1-99 (black) and shNME1-183 (red) to control SK-N-BE(2) (blue) were 1.3e-2 and 2.6e-2.

To evaluate the effects of NME1 depletion on other neuroblastoma cell behaviors, parental neuroblastoma cells and cells with depleted NME1 were monitored for migration into a scratch wounds, using continuous live cell imaging. NME1 depletion resulted in significantly more rapid migration of cells into scratch wounds, with significantly reduced wound width observed at approximately 24 h (Figure 5A,B), consistent with the observed role of NME1 in adult cancer metastases.

**Figure 6:**
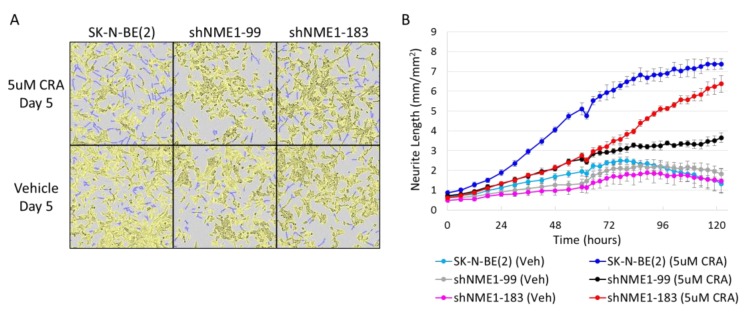
Association of NME1 with neuroblastoma differentiation. Control SK-N-BE(2) cells and cells with depleted NME1 (shNME1-99, shNME1-183) were treated with 5 µM 13-*cis*-retinoic acid (CRA) or vehicle alone and analyzed using continuous live cell imaging with NeuroTrack^TM^ software. (**A**) Imaging obtained of cells after 5 days of vehicle or CRA treatment, with cell bodies in yellow and neurite extensions mapped in blue. (**B**) Total neurite length in parental neuroblastoma cells and in those with depleted NME1 per high-power field were plotted over time. The SK-N-BE(2), the depleted SK-N-BE(2) shNME1-99, and the depleted SK-N-BE(2) shNME1-183 present respective values of *p* = 1.73e-12, *p* = 2.5e-06, and *p* = 6.5e-09 between vehicle and CRA treatment.

Treatment of neuroblastoma tumors frequently results in tumor differentiation, and the differentiation agent 13-*cis*-retinoic acid is a component of standard therapy for children with high-risk neuroblastoma. The mechanisms underlying neuroblastoma cell differentiation, however, are poorly understood. To evaluate the effects of NME1 depletion on neuroblastoma cell differentiation, parental neuroblastoma cells and neuroblastoma cells with depleted NME1 were monitored for differentiation using continuous live cell imaging to determine total neurite length. NME1 depletion resulted in significantly reduced neuroblastoma cell differentiation in response to 13-*cis*-retinoic acid (Figure 6A,B).

### 2.4. Interacting Partners of NME1 and Possible Signaling in Neuroblastoma

**Figure 7:**
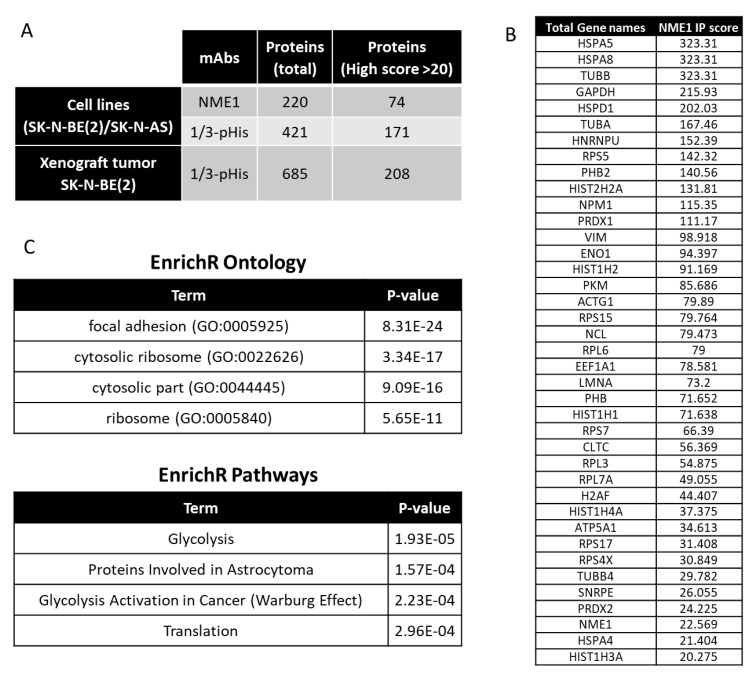
Intracellular pathways associated with NME1 in neuroblastoma. (**A**) Numbers of proteins identified by mass spectrometry from NME1/2 or 1/3-pHis pull-down experiments using a pool of SK-N-BE(2) and SK-N-AS neuroblastoma cell lines or orthotopic xenograft neuroblastoma tumors. (**B**) Neuroblastoma cell lysates were immunoprecipitated using antibodies to NME1 or 1/3-pHis, and isolated proteins were analyzed by mass spectrometry. Proteins identified in all three enrichments with an enrichment score derived from the interaction with NME1 higher than 20 are listed (a complete list and matching comparison of the proteins identified among the three different pull-down experiments are provided as a Supplementary file). (**C**) 59 identified potential NME1 substrates were subjected to pathway analysis using EnrichR (https://amp.pharm.mssm.edu/Enrichr/) [36]. The top four identified pathways (using the Elsevier pathway collection) and gene ontology (using GO_Cellular_Component_2018 section) annotation clusters are listed.

Determination of the functional role of NME1 in neuroblastoma pathogenesis has been limited by the lack of reagents available to evaluate the role of histidine phosphorylation and histidine kinase activity. Furthermore, the full scope of the targets of NME1 histidine kinase activity is not well understood. Using separate NME1 and 1/3-pHis immunoprecipitation experiments with lysates from SK-N-BE(2) and SK-N-AS neuroblastoma cell lines and phosphopeptide immunoaffinity purification using a non-acidic approach [34] with tryptic digests of proteins extracted from orthotopic xenograft tumors (Supplementary Data 3), we were able to identify by mass spectrometry a number of candidate NME1 histidine kinase target proteins, based on their association with NME1and their pull down with anti-pHis antibodies (Figure 7A). Over 1072 unique proteins were identified, corresponding to nonredundant identification from the three pull-downs. In addition, of the 220 NME1-interacting proteins identified from the neuroblastoma cell lines, 140 were common to at least one of the pHis pull-down experiments from cell lines or xenograft tumors, and 59 were detected in each of them and therefore used for cluster analyses. From these common identifications, a selection of 39 proteins with significant scores similar or higher to NME1 (score > 20) are listed in Figure 7B. The list of potential substrates in neuroblastoma includes GAPDH, which has previously been shown to undergo histidine phosphorylation [35]. Additional cluster analyses identified enrichment for pathways related to focal adhesions among the candidate NME1 target proteins (Figure 7C), consistent with our observed association of NME1 expression with neuroblastoma cell migration, as well as possible involvement in protein translation and glycolysis.

## 3. Discussion

Despite intensive, multimodal treatment regimens, children with high-risk and relapsed neuroblastoma have poor survival rates [1]. Improved understanding of critical signaling pathways is required to identify new targets for therapeutic development, which could eventually suggest novel treatment combinations to reduce relapse rates and improve survival for children with neuroblastoma.

The association of the chromosome 17q amplification with poor outcomes in neuroblastoma patients is well established and likely involves the concomitant overexpression of multiple oncogenic proteins, such as STAT proteins, BRCA1, MAP3K14/NIK, and GRB2, which are also present in this common region of amplification as illustrated in Figure 1. However, the abundant amount of proteins from the neuroblastoma cells and xenograft tumors demonstrating histidine phosphorylation, observed in Figure 2 and Figure 3, suggests a functional role for the chromosome 17q gene *NME1* and its NME1 histidine kinase activity. The observed associations between specific *NME1* overexpression with other markers of poor patient prognosis such as *MYCN* amplification reinforces our suggestion that histidine-dependent signaling is likely to be involved in neuroblastoma pathogenesis. NME1 has been shown to be involved in normal cell proliferation, differentiation and development, signal transduction, G protein-coupled receptor endocytosis, and regulation of gene expression [37,38,39,40]. NME1 is also required for neural development including neural patterning and cell fate determination [9], which may be related to our observation that *NME1* shRNA knock-down disrupts differentiation of neuroblastoma cells induced by 13-*cis*-retinoic acid (CRA) treatment, as illustrated in Figure 6. The role of NME1 as a suppressor of metastasis has also been suggested by several studies where the depletion of *NME1* enhances tumor metastases in xenograft models [21,22], whereas *NME1* expression is associated with regulation of genes correlated with adult cancer metastases [20]. The data presented in Figure 5 are consistent with these previous observations and would suggest a similar impact in neuroblastoma tumors.

In contrast to adult tumors, elevated *NME1* expression correlates with aggressive neuroblastoma tumor features [23,24,25], and increased *NME1* expression has been identified as a component of a 3-gene expression signature (*NME1*, *CHD5*, and *PAFAH1B1*) most significantly associated with poor neuroblastoma patient outcomes [26]. Consistent with this duality, a dichotomous role of NME1 function has emerged, with both metastasis suppressing and pro-metastasis activities suggested for NME1 [41,42]. However, the functional roles of NME1 in pediatric cancer in general and in neuroblastoma pathogenesis in particular have not been defined. Yet, a range of expression levels could explain biphasic functions as well as suggest that a basal level of expression is possibly required to balance its catalytic activity in normal cells.

Increased *NME1* gene copy number has been directly identified in 14%–23% of neuroblastoma tumors [43,44], and increased *NME1* expression has also been identified in high-risk neuroblastoma tumors [43,44,45,46,47], possibly secondary to MYCN-mediated regulation of *NME1* expression [48]. Prior studies have also identified a serine/glycine NME1 mutation (S120G) in 21% of a small cohort of advanced neuroblastoma tumors, but not in any low-stage tumors [49], and mutant NME1^S120G^ was shown to abrogate the inhibitory effect of exogenously expressed NME1 on breast carcinoma cell motility [50]. However, subsequent whole exome and whole genome studies of neuroblastoma tumors have failed to identify significant numbers of *NME1* mutations in neuroblastoma [51], and the functional significance of this *NME1*^S120G^ mutation in neuroblastoma pathogenesis has not been defined.

NME1 has been proposed to have a number of enzymatic functions and can act as a nucleoside diphosphate kinase (NDPK) by catalyzing the transfer of the γ-phosphate from nucleoside triphosphates to nucleoside diphosphates in the synthesis of nucleoside triphosphates other than ATP [52,53,54]. NME1 has also been shown to have serine-threonine kinase activity, geranyl and farnesyl pyrophosphate kinase activity, and a 3′-5′ exonuclease activity [55,56,57,58]. NME1 was found to have histidine kinase activity, catalyzing the transfer of an activated phosphate from the autophosphorylated histidine 118 residue (H118) onto target proteins [27]. Although histidine phosphorylation is widely used for bacterial signal transduction, the functional roles of histidine phosphorylation in higher eukaryotes are less well understood [28]. The identification of histidine phosphorylation sites has been limited by the lack of reagents to detect phosphorylated histidine residues, and only a few histidine kinase substrates have been conclusively defined [52,59,60,61,62,63,64,65]. The use of specific 1- and 3-phosphohistidine (1-pHis, 3-pHis) monoclonal antibodies, developed in the Hunter laboratory at the Salk Institute [28,33], allows us to identify potential NME1 targets and downstream signaling pathways.

Using these antibody reagents, Fuhs and colleagues identified 786 potential histidine kinase protein targets by protein immunoaffinity purification using 1- and 3-pHis monoclonal antibodies [33]. Of these proteins, 280 and 156 were found to be exclusive to 1-pHis and 3-pHis antibody purifications, respectively, although the relative significance of the 1-pHis and 3-pHis isoforms remains unknown. Of the proteins identified previously, 142 of the 171 high-score proteins were common to the pHis antibody enriched proteins from neuroblastoma cell lines, along with 70 of the 74 high-score proteins common to the NME immunoprecipitate. In addition, 135 of the 208 high-score proteins were also common to the pHis immunoprecipitate from neuroblastoma xenograft tumors. However, the previous experimental results were based on SILAC scores and the experiments were performed in different types of cells, and further detailed explorations of potential histidine kinase targets are clearly needed.

The functional role of histidine kinases and histidine phosphorylation in neuroblastoma tumors remains undefined. However, the inverse correlations of NME1 and the recently identified histidine phosphatase LHPP with neuroblastoma patient outcomes suggests a significant role for the regulation of histidine phosphorylation in neuroblastoma pathogenesis. Furthermore, the expression of NME1 and LHPP were inversely correlated in neural tissues such as the brain and adrenal gland using data from the human protein atlas (Supplementary Data 4).

NME1 is a member of the NME protein family, with 10 NME family genes identified to date in humans encoding full-length, tandemly repeated NM23 domains, or, in one case, a truncated NM23 domain [66] present in all eukaryotes. NME1 is a member of the first group of NME proteins (NME1-4), which share 58%–88% overall homology, with NME1 and NME2, sharing 88% sequence homology [28]. NME1 generally functions as a hexamer composed of both ‘A’ (encoded by NME1) and ‘B’ (encoded by NME2) isoforms [67], and this homology combined with colocalization suggests potential overlapping functions and possibly explains the slight increase in *NME2* expression after *NME1* knock-down (Figure 4b). Considering that *NME2* is also overexpressed due to the chromosome 17q amplification in these cells, this observation could also sustain the idea that a defined range of NME1 and NME2 expression is required for its functional impact. The specific functional role of NME2 in neuroblastoma, however, remains unknown. Individual NME1 and NME2 knockout mice have been found to be viable, with defective growth but normal lifespans [68,69], while the NME1/2 double knockout results in embryonic lethality in mice, further suggesting that NME1 and NME2 are capable of some degree of functional compensation. Our data demonstrating phenotypic effects of isolated NME1 depletion in neuroblastoma cell lines, however, suggests some unique functional roles of NME1. The relative roles of NME1 and NME2 in these hexamers, in the regulation of histidine phosphorylation, and in neuroblastoma pathogenesis remain undefined.

The high degree of homology and the conservation of the NME gene family of histidine kinases throughout evolution support the idea that histidine phosphorylation has important roles in basic cellular processes as well as disease pathogenesis. A better knowledge of the binding partners and histidine kinase substrates is critically needed to define pathways regulated by histidine phosphorylation and histidine kinase activity. In theory, many of the identified pHis-containing proteins in mammalian cells could be phosphorylated by one or several of the members of the NME family, particularly considering that the different NME family members can have distinct sites of intracellular localization, such as NME4 being localized to mitochondria [70] and NME7 being associated with the centrosome [71]. NME1, NME2, and NME3 were also each identified from the NME1 immunoprecipitated from neuroblastoma cells, which also supports the notion of hetero-oligomerization and the autophosphorylation of these family members.

The cellular functions directly regulated by NME1 expression and histidine kinase activity in neuroblastoma remain to be determined. NME1 was initially identified as a suppressor of tumor metastasis, and recent studies have identified critical intracellular interactions linking NME1 to tumor cell motility and metastatic spread [72,73,74]. Our studies have further identified a potential role for NME1 in neuroblastoma cell migration and differentiation, as demonstrated by the fact that NME1-depleted neuroblastoma cells exhibited more rapid migration into scratch wounds (Figure 5a,b) and reduced CRA-induced differentiation (Figure 6a,b). While NME1 NDPK activity has been shown to contribute to dynamin function and endothelial cell contractility [75,76], the relative role of NME1 histidine kinase activity in the process of tumor cell metastasis remains to be determined. However, numerous proteins identified as potential pHis-containing substrates are involved in cell adhesion and could provide opportunities for further exploration. Limited mutational analyses suggested that histidine kinase activity of NME1 may correlate with suppression of cancer cell motility [50]. Our preliminary studies also have identified other candidate targets of NME1 histidine kinase activity, including ENO1, GAPDH, and PKM, all proteins with potential roles in glycolysis, suggesting that the associations of NME1 expression with neuroblastoma patient outcomes may also be linked to effects on cellular metabolism. Interestingly, the cluster annotation also revealed an association with astrocytoma, a type of brain tumor.

In conclusion, our study further delineates the associations of *NME1* expression with chromosome 17q amplification, tumor prognostic features, and overall patient outcomes in neuroblastoma. We have identified the presence of histidine phosphorylation of a number of proteins, including NME1 and NME2, in neuroblastoma cells and tumors, and have identified potential links between NME1 expression and neuroblastoma cell migration and differentiation. Our results have also generated a list of candidate NME1 kinase protein targets that appear to play roles in cell adhesion and migration.

Our results clearly require further validation, and future studies will need to further evaluate the functional role of NME1 histidine kinase activity in neuroblastoma pathogenesis, including its potential roles in cell migration, metastasis, and differentiation. A better understanding of the specific signaling pathways regulated by NME1 histidine kinase activity and identification and validation of NME1 histidine kinase targets in neuroblastoma are critically needed, which could lead to the identification of new therapeutic targets and the development of novel approaches to neuroblastoma therapy.

## 4. Materials and Methods

### 4.1. Immunoblotting

Immunoblotting with monoclonal antibodies to 1-pHis and 3-pHis and polyclonal pNME1/2-H118 antibodies were performed with modifications to standard procedures to help preserve phosphorylated histidine for detection. Buffers were adjusted to pH 8–9 to stabilize phosphorylated histidine, and methods were modified to avoid heating samples. Protein samples were prepared in pH 8.8 sample buffer (5× = 10% SDS, 250 mM Tris-HCl, pH 8.8, 0.02% bromophenol blue, 50% glycerol, 50 mM EDTA, 500 mM DTT) for electrophoresis. Whole cell lysates were prepared by rinsing 70%–100% confluent 10 cm^2^ dishes twice with 5 mL cold PBS buffer (PBS-/-, pH 8). Cells were scraped directly into 2× pH 8.8 sample buffer and incubated on ice. Cells were disrupted and DNA sheared by either a tip sonicator (3x 10 s bursts / 10 s ice) or passing through a 21G needle 10 times, followed by a 26G needle at least 25 times. Lysates were clarified by centrifugation (14,000× *g* for 5–15 min at 4 °C) and analyzed immediately using freshly prepared Bis-Tris polyacrylamide minigels with a modified, pH 8.8 stacking gel and either 10% or 12.5% resolving gels. Electrophoresis buffer recipes were as follows: Running Buffer: (1× 20 L, pH 8.5) 20 g SDS, 60 g Trizma Base, 288 g glycine, dH2O to 20 L; Transfer Buffer: (1× 4 L, pH 8.5) 56.7 g glycine, 4 g SDS, 12 g Trizma Base, 800 mL MeOH, dH2O to 4 L. All electrophoresis steps were performed at 4 °C and samples were resolved at 90–100 V for 2–4 h. Proteins were transferred to PVDF or Immobilon-FL PVDF membranes at 30 V for 12–18 h at 4 °C and immediately incubated for 45–60 min at room temperature or at least 2 h at 4 °C in Casein Blocking Buffer (0.1% casein, 0.2× PBS -/-). Primary antibodies were diluted in blocking buffer with 0.1% Tween-20 and then incubated with membranes for 1 h at room temperature or 3–18 h at 4 °C. Membranes were washed three times for 5 min each with 0.1% TBST before incubation with secondary antibodies for 45 min at room temperature, then washed three times for 5 min each with 0.1% TBST. Immunoblots were imaged on a LI-COR Odyssey Infrared Imaging System. For rabbit antibodies, Alexa Fluor^®^ 680 goat anti-rabbit IgG secondary antibodies were diluted 1:20,000 in blocking buffer supplemented with 0.1% Tween-20 and 0.01% SDS. Goat anti-mouse IgG secondary antibody (DyLight 800 conjugate) was diluted 1:20,000 in blocking buffer supplemented with 0.1% Tween-20 and 0.01% SDS and incubated alone or co-incubated with Alexa Fluor^®^ 680 goat anti-rabbit secondary antibodies if needed. Rabbit monoclonal anti-pHis (Hunter Lab, La Jolla, CA, USA, Salk—hybridomas SC1-1 and SC44-1) and polyclonal anti-pNME1/2 (Hunter Lab, La Jolla, CA, USA, Salk—#7395-8) were used at 0.5 µg/mL. Antibodies to NME1/2 (Cell Signaling 3345) and LHPP (NBP1-83273) were used at 1:1000; antibodies to β-actin (Sigma A5316) were used at 1:20,000.

### 4.2. Cell Culture and Stable Cell Line Generation

Neuroblastoma cell lines SK-N-AS, SK-N-SH, SK-N-BE(2) and IMR32 were obtained from ATCC and maintained in RPMI-1640 supplemented with 10% FBS, 1 mM sodium pyruvate, 2 mM L-glutamine, 1000 µg/mL streptomycin, 1000 IU/mL penicillin, and 2.5 µg/mL amphotericin at 37 °C in 5% CO_2_. Stable knockdown of *NME1* in SK-N-SH and SK-N-BE(2) cells was performed using Lipofectamine^®^ 2000 (Invitrogen, Carlsbad, CA, USA) to transfect 2.5 µg of shRNA clone 99 and clone 183 (TRCN0000010062, TRCN0000010055, Sigma, St. Louis, MO, USA) in separate 35 mm plates. Three days post transfection, 2 µg/mL puromycin was added and maintained for three weeks to select for stable transfectants. *NME1* knockdown was confirmed by quantitative PCR and immunoblot.

### 4.3. Quantitative PCR

RNA was isolated (Qiagen RNeasy Kit) from parental SK-N-BE(2) cells and cells with depleted NME1 (shNME1-99, shNME1-183), reverse transcribed to cDNA (Applied Biosystems High Capacity cDNA Reverse Transcription Kit), and quantitative PCR was performed using sequence specific primers for human *NME1* (5′-AAGGAGATCGGCTTGTGGTTT-3′; 5′-CTGAGCACAGCTCGTGTAATC-3′), human *NME2* (5′-GGACTTCTGCATTCAGGTTGGC-3′; 5′- TGTAGTCAACCAGTTCTTCAGGC-3′), and as a control human *GAPDH* (5′- GTCTCCTCTGACTTCAACAGCG-3′; 5′-ACCACCCTGTTGCTGTAGCCAA-3′). Fold change of *NME1* and *NME2* in cells with depleted NME1 was calculated relative to *GAPDH* and to *NME1* and *NME2* of non-transfected parental SK-N-BE(2) cells.

### 4.4. Cell Proliferation

Cell proliferation was measured using continuous live cell imaging with the IncuCyte Zoom^TM^ Live-Cell Analysis System (Essen BioScience, Ann Arbor, MI, USA) to measure confluence over time. Cells were plated at 5000 cells per well in a 96-well plate with 6 replicates per experiment, and phase contrast images were taken using a 10× objective every 6 h over 8 days. Percent cell confluence and standard deviations were calculated using the IncuCyte Analysis software. 

### 4.5. Scratch Wound Cell Migration

Cell migration was evaluated using the IncuCyte Zoom^TM^ 96-Well Scratch Wound Cell Migration assay. Cells were seeded at 100,000 cells per well in a 96-well ImageLock tissue culture plate (Essen BioScience, Ann Arbor, MI, USA) with 12 replicates per experiment and incubated overnight at 37 °C in 5% CO_2_ before making identical individual scratches in each well using the Wound Maker tool (Essen BioScience, Ann Arbor, MI, USA). Wells were gently washed twice with culture media to remove dislodged cells and cell culture media was replaced. The assay plate was placed in the IncuCyte Zoom^TM^, and phase contrast images using a 10× objective were taken every 3 h for 48 h. Wound width was calculated using the IncuCyte scratch wound analysis software. 

### 4.6. Cell Differentiation

Neurite outgrowth was analyzed using the IncuCyte Zoom^TM^ live cell imaging system (Essen BioScience, Ann Arbor, MI, USA) and NeuroTrack^TM^ Software. Cells were seeded at 2000 cells per well and incubated overnight at 37 °C in 5% CO_2_. Cells were treated with 5 µM 13-*cis*-retinoic acid or 0.1% dimethyl sulfoxide (vehicle) daily for 8 days, with 6 independent replicates per treatment. Phase contrast images were taken every 6 h using a 10× objective. Total neurite length per high-power field was calculated with the IncuCyte NeuroTrack^TM^ Software and was normalized to the image area (mm summed length/mm^2^ image area).

### 4.7. Immunofluorescence Staining

1-pHis, 3-pHis, and NME1/2 were detected in SK-N-SH cells by immunofluorescence staining employing a non-acidic method [77]. Cells were either plated on cover slips and grown to 50% confluence or suspended in OCT, pipetted into a cryomold, frozen on dry ice, and 5-micron sections were cut and dried to a charged glass slide. Coverslips/slides were washed twice with ice-cold PBS (pH 8.5), fixed with 8% paraformaldehyde (pH 8.5) for 15 min at room temperature, and washed 3 times for 5 min with 4 °C PBS (pH 8.5) (control slides and coverslips were washed with 90 °C PBS at pH 4.0). Cells were permeabilized with 0.1% Triton X-100 in PBS (pH 8.5) for 15 min at room temperature, then washed 3 times for 5 min with 4 °C PBS (pH 8.5) (control slides and coverslips were washed with 90 °C PBS at pH 4.0), and washed again 1 time with 1% bovine serum albumin (BSA) in Tris-buffered saline with 0.1% Tween-20 (TBST) (pH 8.5) for 5 min at room temperature. Cells were blocked in 10% BSA in TBST (pH 8.5) at room temperature for 30 min before incubating with primary antibody diluted in 1% BSA in TBST (pH 8.5) for 2 h at room temperature, then washed 3 times for 5 min with TBST (pH 8.5) at room temperature. Cells were incubated in secondary antibodies diluted 1:500 in 1% BSA in TBST (pH 8.5) for 1 h at room temperature, washed 3 times with TBST (pH 8.5) at room temperature, then mounted with Fluoromount Aqueous mounting medium (F4680, Sigma, St. Louis, MO, USA). 1-pHis SC1-1, and 3-pHis SC44-1 antibodies (Hunter Lab, La Jolla, CA, USA) were diluted to 2.5 µg/mL, NME1 antibodies (3345, Cell Signaling Technologies, Danvers, MA, USA) were diluted 1:100 and mixed with NME2 antibodies (131329, Abcam, Cambridge, United Kingdom) diluted to 1.25 µg/mL. Secondary goat anti-rabbit IgG antibody Alexa Fluor^®^ 568 (A-11036, Invitrogen, Carlsbad, CA, USA) was diluted to 4 ug/mL and mixed with DAPI (D1306, Fisher, Waltham, MA, USA) diluted to 10 µg/mL. Images were taken on the Keyence BZ-X710.

### 4.8. Protein Immunoprecipitation from Neuroblastoma Cell Lines for MS/MS

Proteins were extracted from 10 × 10^6^ SK-N-BE(2) and SK-N-AS neuroblastoma cells using cold lysis buffer (20 mM Tris HCl pH8, 100 mM NaCl, 2 mM EDTA, 1% NP40, phosphatase inhibitor (PhoSTOP), complete EDTA-free Protease Inhibitor (Roche, Basel, Switzerland) and 2.5 U/µL Benzonase) for 30 min at 4 °C. Lysates were clarified by centrifugation (20,000× *g* for 20 min) and incubated with 50 mg of protein A agarose beads at 4 °C for 1 h. Supernatant was collected after centrifugation (2500 rpm for 3 min at 4 °C) and 2 µg of antibodies (mouse monoclonal nm23-H1 sc-465 antibodies from Santa Cruz or rabbit monoclonal 1-pHis SC1-1 and 3-pHis SC44-1 antibodies from the Hunter lab) was added before incubation with 50 mg of protein A agarose beads for 1 h at 4 °C using constant rotation. Beads were isolated by centrifugation (5000 rpm for 1 min at 4 °C) and washed twice with 1 mL of 20 mM Tris HCl pH 8, 100 mM NaCl, 2 mM EDTA, and 0.1% NP40, then with 1 mL of 20 mM Tris HCl pH 8, 100 mM NaCl, and 2 mM EDTA. 

Proteins were eluted with at least 3 volumes of urea 8 M (pH 10), 20 mM Tris, and 100 mM NaCl while under agitation (1000 rpm thermomixer) for 30 min at room temperature. Proteins were reduced and alkylated with 10 mM DTT and 10 mM chloroacetamide (C0267, Sigma, St. Louis, MO, USA) for 30 min at room temperature. Urea was diluted approximately four-fold (~2 M) with Tris HCl 20 mM pH 8.5 before the addition of trypsin (~5 µg). Lysates were incubated overnight at 4 °C. Peptides were lyophilized and stored at −20 °C.

### 4.9. Phosphopeptide Immunoaffinity Purification from Xenograft Tumors for MS/MS

Mouse experiments were authorized through the IACUC approval protocol number S16208 (date approved: 08/22/2019; date expires: 08/22/2022) and the UCSD Animal Welfare Assurance Number A3033-01.

SK-N-BE(2) xenograft tumors from mice were frozen and pulverized in liquid nitrogen and solubilized with denaturing buffer (urea 8 M pH 10, Hepes 20 mM, phosphatase inhibitors (PhosSTOP), Octyl-B-D-Glucopyranoside 30 mM, and NH_4_HCO_3_ 1 M) on ice. Lysates were passed through a 20G syringe (3–5 times), then sonicated (3–5 periods of 10 s bursts/10 s on ice). Then, 500 U/mL of benzonase was added to the lysate and supplemented with 1 mM MgCl_2_, 10 mM DTT, and 10 mM of chloroacetamide (C0267, Sigma, St. Louis, MO, USA) for reduction and alkylation at 37 °C for 1 h. The lysate was centrifuged at 4000 g for 15 min at room temperature. Proteins from the supernatants were precipitated with at least 8 volumes of cold methanol to one volume of sample supplemented with one volume of chloroform. The solution was vortexed and incubated for at least 1 h at −20 °C, before centrifugation at >7500 g for 30 min at 4 °C. Impurities were removed, and the tube and pellet were washed with 50 mM NH_4_HCO_3_ (pH 8.5). Pellets were resuspended in 50 mM NH_4_HCO_3_ (pH 8.5) with the phosphatase inhibitor mix (PhosSTOP, Roche). Protein concentrations were measured with a Bio-Rad DC protein assay, adjusting volumes to ensure protein concentrations of less than ~10 mg/mL. About 8 mg of proteins were digested overnight with trypsin at 1/50 *w*/*w* at room temperature. Samples were then centrifuged for 5 min at 20,000 g and supernatant stored at 4 °C.

Immunoaffinity purification (IAP) columns, containing one volume of protein A crosslinked with a mixture of three specific anti-1-pHis monoclonal antibodies (SC1-1, SC50-3, SC77-11) and three specific 3-pHis monoclonal antibodies (SC39-6, SC44-1, SC56-2), were equilibrated three times with one volume of NH_4_HCO_3_ (pH 8.5) at room temperature without drying out the protein A [34]. Ten minutes before placing the sample on the IAP column, 100 µM of TLCK (Tosyl-L-lysyl-chloromethane hydrochloride, Trypsin inhibitor T7254; Sigma) was added to the peptide solution at room temperature. While collecting the flow through, peptides were passed over the column twice and then washed five times with one volume of 50 mM NH_4_HCO_3_ (pH 8.5). Phosphopeptides were eluted twice with one volume of 100 mM TEA pH 11. Elution samples were dried using a speed-vacuum and stored at −80 °C.

### 4.10. LC-MS/MS

Peptide samples were resuspended with cold buffer A (5% ACN, 95% H_2_O, 0.1% formic acid, pH 2) on ice, prior to desalting/loading under high pressure using Aqua-C18 5 µm resin (3 cm) within a 250 µm column (fused silica capillary with a Kasil frit). The same C18 resin (12 cm) was used for a 100 µm analytical column. Nano liquid chromatography tandem mass spectrometry (nano-LC-ESI MS/MS) was performed in positive ion mode using an Agilent 1200 G1311 Quaternary Pump coupled to an LTQ-Velos Orbitrap. 

A 120-min elution gradient was used: 10% Buffer B (80% ACN, 20% H_2_O, 0.1% formic acid, pH 2) for 5 min, 40% Buffer B for 80 min, to 100% Buffer B for 100 min continuing to 110 min. One cycle consisted of one full scan mass spectrum (m/z 300–1600) with a maximum injection time of 10 ms at 60,000 resolution followed by up to 20 data-dependent collision-induced dissociation (CID) MS/MS spectra. Application of mass spectrometer scan functions and HPLC solvent gradients were controlled by the Xcalibur data system.

## Supplementary Materials

Supplementary materials can be found at https://www.mdpi.com/1422-0067/21/9/3319/s1.

## Abbreviations

ACN	Acetonitrile
ATCC	American Type Culture Collection
CID	Collision Induced Dissociation
CRA	13-cis-Retinoic Acid
EDTA	Ethylenediaminetetraacetic acid
EFS	Event-Free Survival
HCl	Hydrochloric acid
HPLC	High-Performance Liquid Chromatography
LC	Liquid Chromatography
LHPP	Lysine-Histidine-Pyrophosphate Phosphatase
MS	Mass Spectrometry
NDPK	Nucleoside Diphosphate Kinase
NH_4_HCO_3_	Ammonium Bicarbonate
OCT	Optimal Cutting Temperature
OS	Overall Survival
PVDF	Polyvinylidene Difluoride
Q-PCR	Quantitative Polymerase Chain Reaction
SC	Subclone
SDS	Sodium Dodecyl Sulfate
SEQC	Cell Sequencing
TEA	Triethanolamine
TLCK	Tosyl-L-Lysyl-Chloromethane Hydrochloride
