# Supplementary material for: The Potential Functional Roles of NME1 Histidine Kinase Activity in Neuroblastoma Pathogenesis
Source: Int J Mol Sci. 2020 May 7;21(9):3319. doi: 10.3390/ijms21093319 (PMC7247550; doi:10.3390/ijms21093319)
Supplement: Supplementary file 1 [file ijms-21-03319-s001.zip › ijms-778301 Supplementary Data final.pdf]

International Journal of *Molecular Sciences*

Supplementary Data 1

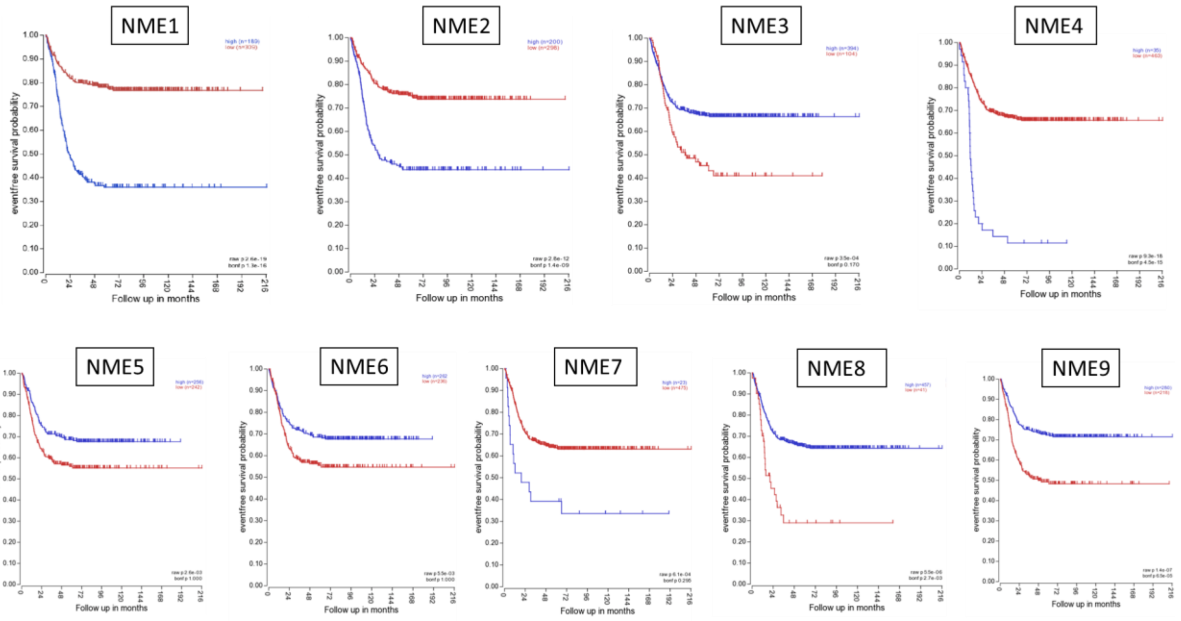

Using R2 Genomics Analysis and Visualization Platform, neuroblastoma patients were divided into high (blue) and low (red) genes expression groups for each of the nine *NME* family members *(NME1-9)* and survival curves were generated. Event-free survival (EFS) are shown with patient numbers in parentheses.

Supplementary Data 2

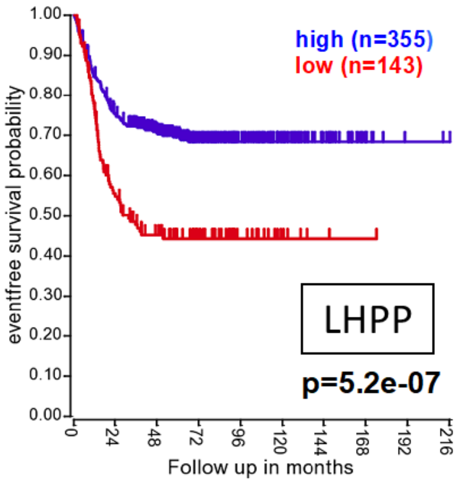

Using R2 Genomics Analysis and Visualization Platform, neuroblastoma patients were divided into high (blue) and low (red) LHPP gene expression groups and survival curves were generated. Event-free survival (EFS) are shown with patient numbers in parentheses.

Supplementary Data 3

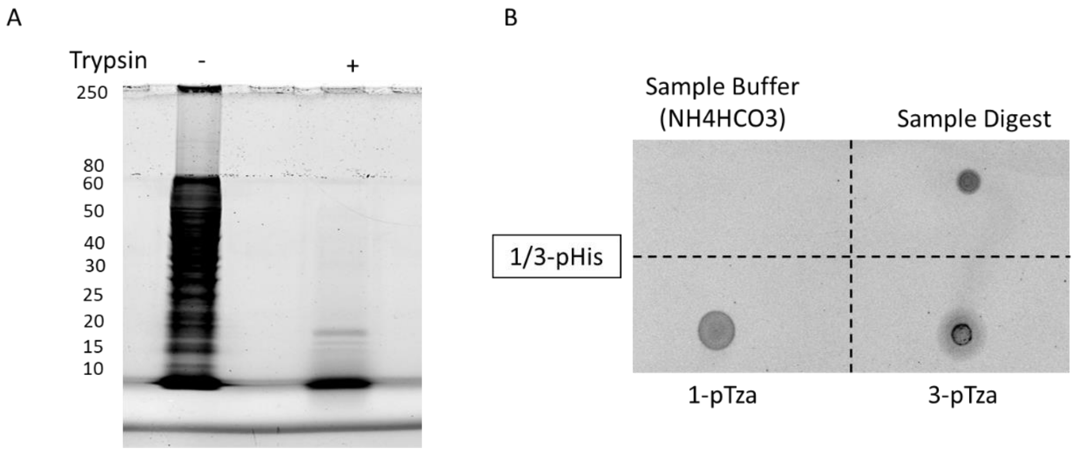

(**a**) Coomassie staining (**b**) Dot-blot stained with 1/3-pHis mAbs. Sample digest from SK-N-BE(2) orthotopic xenograft tumor (8 µg). 1-pTza and 3-pTza peptides (50 ng).

Supplementary Data 4

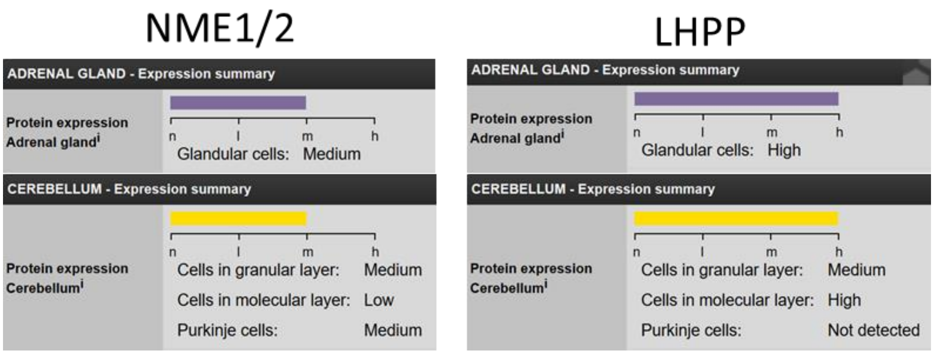

Protein expression range per tissue specificity available from http://www.proteinatlas.org
